# Deodorizing the Breath and Cleansing the Teeth

**Published:** 1862-08

**Authors:** 


Deodorizing the Breath and Cleansing the Teeth.—We find the following formulæ, which may prove occasionally convenient, in the *Druggist's Circular*. It is important that the hypochlorite of lime employed should be fresh and good, coming up to the standard of the Pharmacopœia:

*Dr. Angelot's Preparations.*—The following preparation has been employed by Dr. Angelot, of Briancon, in the treatment of ulceration of the gums, a very frequent complaint with soldiers: Take of hypochlorite of lime, from 10 to 25 grains; mucilage of gum-arabic, 1½ to 4 drachms; syrup of orange-peel, 1½ to 2 drachms. Mix thoroughly. This mixture is employed as a lotion to the ulcerated gums.

*Pastilles of Hypochlorite of Lime*—Several formulas for the preparation of the pastilles have been successively published; these preparations have the advantage over those which are liquid, of being more easily transported.

*First Formula.*—Take of hypochlorite of lime, 7 drachms; sugar, flavored with vanilla, 3 drachms; gum-arabic, 5 drachms. The pastilles are made *secundum artem*, so as to weigh from 10 to 11 grains. Two or three of these pastilles are sufficient to remove from the breath the disagreeable odor produced by tobacco smoke. The pastilles thus prepared have a gray color, and become quite hard; if pastilles of a white color are required, the following substances are employed:

*Second Formula.*—Take of dry hypochlorite of lime, 20 grains; pulverized sugar, 1 oz.; gum tragacanth, 16 grains. The hypochlorite of lime is triturated in a glass mortar, and a small quantity of water is poured upon it; it is then left to repose, decanted, and a second quantity of water added; the two liquids are filtered, and the gum and sugar added so as to form a paste. This is divided into pastilles, weighing from 12 to 16 grains. If it is desired to aromatize the paste, one or two drops of any essential oil may be added; the oil should be added to the sugar and gum before the paste is formed.

*Deschamps' Pastilles.*—Take of dry hypochlorite of lime, 2 drachms; sugar, 8½ ounces; starch, 8 drachms; gum tragacanth, 1 drachm; carmine, 2½ grains. The pastilles should be made so as to weigh about 2½ grains; five or six may be taken in the space of two hours. By employing starch in the preparation of the lozenges, Deschamps wishes to prevent the yellow color which they would otherwise assume.

*Deschamps' Dentifrice for removing the Yellow Color from the Teeth.*—Take of dry hypochlorite of lime, ½ drachm ; red coral, 2 drachms. Triturate well and mix thoroughly. This powder is employed in the following manner : a new brush is slightly moistened, then dipped in the powder and applied to the teeth. According to the author, a few days' use of this powder will produce a marked alteration in the appearance of the teeth, which will acquire a white color.—*Chicago Medical Journal.*

